# Regulating AMPA Receptors with Isoxazole-4-Carboxamide Derivatives: An Electrophysiological Study

**DOI:** 10.3390/jox15020040

**Published:** 2025-03-08

**Authors:** Mohammad Qneibi, Mohammed Hawash, Sosana Bdir, Mohammad Bdair, Tala Idais, Iyas Sarhan, Joud Touqan

**Affiliations:** 1 Department of Biomedical Sciences, Faculty of Medicine and Health Sciences, An-Najah National University, P400 Nablus, Palestine; s12027767@stu.najah.edu (S.B.); s12143520@stu.najah.edu (M.B.); s12220051@stu.najah.edu (T.I.); s12113103@stu.najah.edu (I.S.); joudttouqan@gmail.com (J.T.); 2 Pharmaceutical Chemistry and Technology Division, Faculty of Pharmacy, An-Najah National University, P400 Nablus, Palestine; mohawash@najah.edu

**Keywords:** chronic pain, AMPA receptor, isoxazole–carboxamide, GluA2, desensitization, deactivation

## Abstract

Isoxazole carboxamide derivatives are intriguing modulators of ionotropic glutamate receptors; more specifically, their prospective analgesic activities based on non-opioid pathways have sparked widespread research. α-amino-3-hydroxy-5-methyl-4-isoxazolepropionic acid (AMPA) receptors, especially Ca^2+^-permeable subtypes that are highly expressed in the spinal dorsal horn, play a critical role in nociceptive transmission and inflammatory pain. Herein, the neuromodulatory effects of these derivatives on AMPA receptor activity have been studied, focusing on their potential as modulators of AMPA receptors, a target implicated in pain and neurological disorders. The whole-cell patch clamp technique for electrophysiological recordings was used to investigate the effect of twelve isoxazole-4-carboxamide derivatives (CIC-1-12) on AMPA receptors’ whole-cell currents and kinetics, including deactivation and desensitization. The isoxazole-4-carboxamide derivatives tested as inhibitors of AMPA receptor activity were very potent, with an 8-fold inhibition by CIC-1 and a 7.8-fold reduction by CIC-2. Additionally, these compounds profoundly altered the biophysical gating properties of both homomeric and heteromeric receptor subunits. These findings emphasize the therapeutic promise of isoxazole-4-carboxamide derivatives due to their potential as AMPA receptor modulators. Their ability to affect receptor activity and gating properties makes them promising candidates for future treatments for controlling pain.

## 1. Introduction

One of the most common conditions encountered in clinical medicine is chronic inflammatory pain, which seriously affects the quality of life for patients and presents a substantial burden to health resources on a worldwide scale [1,2]. Chronic inflammatory pain refers to painful conditions characterized by continuous nociceptor activation caused by persistent inflammatory reactions that can be clinically expressed in states such as rheumatoid arthritis, inflammatory bowel disease, and neuropathic chronic painful conditions [3,4]. The World Health Organization estimates that about 20% of the world’s population suffers from chronic pain, and inflammatory pain constitutes a large portion of these [5]. The economic burden is enormous, with an estimated annual cost of USD 635 billion in the United States alone, surpassing the combined costs of cancer, diabetes, and heart disease [6].

While several pharmacological options exist, the management of chronic inflammatory pain remains unsatisfactory [7,8]. Current strategies, which are predominantly based on opioids and nonsteroidal anti-inflammatory drugs (NSAIDs), have considerable limitations [9]. Opioids, while effective in the setting of acute pain, are burdened with addiction, tolerance, and severe side effects such as respiratory depression. NSAIDs, though in widespread use, carry risks of gastrointestinal bleeding, cardiovascular complications, and limited efficacy in managing severe or long-standing pain [10]. These challenges emphasize the urgent need for innovative therapeutic strategies that address the etiology underlying chronic inflammatory pain [11].

At the core of pain signaling and mediating synaptic transmission in the central nervous system (CNS) is glutamate [12]. Glutamate exerts its main effects through ionotropic glutamate receptors, among which α-amino-3-hydroxy-5-methyl-4-isoxazolepropionic acid (AMPA) receptors play a very important role. AMPA receptors are tetrameric assemblies composed of subunits GluA1–GluA4 and, due to their unique configuration, exert diverse functional properties, including ion selectivity and receptor trafficking [13,14]. Recently, AMPA receptor dysregulation has increasingly been implicated in the pathophysiology of chronic pain. More specifically, overactivation of AMPA receptors has been implicated in central sensitization, wherein the CNS amplifies the pain signal, leading to heightened sensitivity and prolongation of pain perception [15,16]. Experimental studies have shown that genetic deletion or pharmacological inhibition of selected AMPA receptor subunits, such as GluA1 or GluA2, reduces pain hypersensitivity in preclinical models [17]. Spinal AMPA receptor-dependent plasticity has also been suggested to be a mechanism of dorsal horn pathway sensitization—a proposed mechanism underlying chronic inflammatory pain [18]. These data position AMPA receptors as a potent target for therapeutic intervention [19,20,21].

Overactivation of AMPA receptors plays a pivotal role in chronic inflammatory pain by amplifying excitatory neurotransmission, leading to central sensitization [22]. This heightened excitatory state prolongs pain perception and reduces the effectiveness of conventional analgesics [23]. Our hypothesis is that the selective modulation of AMPA receptor kinetics through isoxazole-4-carboxamide derivatives can counteract this pathological process while preserving the normal synaptic function. Specifically, these derivatives act as negative allosteric modulators, binding to regulatory sites distinct from the glutamate-binding domain. This interaction results in a dual effect: (1) prolonging deactivation by stabilizing receptor closure after glutamate unbinding, thereby reducing excessive post-synaptic excitatory currents and (2) accelerating desensitization, which shortens receptor activation during prolonged glutamate exposure, preventing excitotoxicity and nociceptive amplification. Unlike direct antagonists, these compounds do not fully block AMPA receptor activity, ensuring that physiological transmission remains intact while preventing pathological overactivation [24].

Recently, numerous studies have been conducted on heterocycles such as isoxazole, pyrazole, and thiazole derivatives as pharmacologically active agents [25,26,27,28,29,30,31]. Isoxazole derivatives represent a class of compounds with a common structure in the isoxazole ring that has attracted attention because of a wide range of pharmacological activities [32] including anticancer [33,34,35], hypoglycemic [36], antiviral [37], antimicrobial [38,39], anti-inflammatory [40,41], analgesic [38], and antioxidant activities [32]. Such observations make them fascinating molecules for pharmaceutical development. Isoxazole derivatives bearing a carboxamide group have represented one of the most active research topics in the literature. The isoxazole scaffold combined with a carboxamide and other functional scaffolds, such as 3-chloro-2,4-dimethoxyphenyl and methylthiophenyl groups, confers enhanced AMPA receptor activity [42]. This is corroborated by several preclinical studies showing that some isoxazole derivatives can modulate the expression of AMPA receptor subunits and prevent excitotoxicity and central sensitization. It is also based on the research that AMPA receptor targeting with these derivatives can offer extra boosts compared to the therapies that are currently used for treatment [43]. For example, there is a possibility of effectively relieving pain by the selective modulation of AMPA receptor activities, along with reducing the side effects that accompany the use of opioids or NSAIDs.

This study investigates previously published isoxazole-4-carboxamides to elucidate their potential as modulators of AMPA receptors, a target implicated in chronic pain [27]. By examining the effects of these compounds on AMPA receptor activity, deactivation, and desensitization, and by investigating the structural determinants of their interactions with the receptor, this research aims to elucidate the mechanisms of AMPA receptor modulation and their relevance to pain pathways. This work contributes valuable in vitro data towards the development of novel therapeutic strategies targeting AMPA receptors, a key player in pain pathophysiology.

While this study focuses on in vitro mechanisms, the broader context of chronic pain management motivates this research. Chronic pain, including inflammatory pain, represents a significant global health challenge. AMPA receptors, particularly those in the spinal dorsal horn, are crucial for synaptic plasticity and sensitization processes that underlie chronic pain states. Therefore, understanding how to modulate AMPA receptor activity is a critical step in developing potential therapeutic interventions. This study aims to contribute to understanding of that by providing a characterization of the interaction between isoxazole-4-carboxamide derivatives and AMPA receptors. The insights gained from this research may inform the design of future compounds with optimized properties for targeting AMPA receptors in the context of chronic pain.

## 2. Materials and Methods

### 2.1. Chemical Caracterization and Analysis 

All chemicals were purchased from Alfa Aesar (Heysham, England, UK) and Sigma-Aldrich (Darmstadt, Germany). The melting points of the compounds were measured using an SMP-II Digital Melting Point Apparatus, with no corrections applied. Nuclear Magnetic Resonance (NMR) spectra, including both ^1^H-NMR and ^13^C-NMR, were obtained in DMSO-d6 using a Bruker Avance III 500 MHz High-Performance Digital FT-NMR spectrometer (Bruker Corporation, Billerica, MA, USA) located at the Department of Chemistry, Faculty of Science, University of Jordan, Jordan. Chemical shifts are reported in delta (δ) values in parts per million (ppm). High-resolution mass spectrometry (HRMS) data were acquired with a Waters LCT Premier XE Mass Spectrometer (waters Corporation, Milford, MA, USA), a high-sensitivity orthogonal acceleration time-of-flight instrument. The HRMS analyses were performed using the ESI (+) ionization technique with the instrument connected to an EQUITY Ultra Performance Liquid Chromatography system at the Faculty of Pharmacy, Gazi University, Ankara, Turkey.

#### Synthesis of Isoxazole–Carboxamide Derivatives

All the tested derivatives were synthesized before except the CIC-11 compound. The synthesis of 3-(2-chloro-6-fluorophenyl)-5-methyl-N-[4-(trifluoromethoxy) phenyl]-1,2-oxazole-4-carboxamide CIC-11 was performed as follows: carboxylic acid was dissolved in dichloromethane (DCM) at room temperature and was then treated with dimethylaminopyridine (DMAP), and after 30 min, N-(3-dimethylaminopropyl)-N′-ethylcarbodiimide hydrochloride (EDC) was added. To the reaction mixture, an aniline derivative was added after 30 min and stirred at ambient temperature under an argon atmosphere for 48 h. The reaction was followed by TLC until completion. Excess aniline was removed by extraction with diluted HCl. The resulting mixture was then concentrated under reduced pressure using a rotary evaporator and purified through column chromatography with an appropriate solvent system. The purity of the title compounds was further checked by ultra-performance liquid chromatography, which showed >97% in each case for the purity. The synthesis of the other isoxazole–carboxamide derivatives and its corresponding spectral data have been described earlier [44]; the spectrum data can be found in the Supplementary Materials.

3-(2-chloro-6-fluorophenyl)-5-methyl-N-(4-(trifluoromethoxy)phenyl)isoxazole-4-carboxamide (CIC-11)

^1^H NMR (400 MHz, DMSO_6_) δ: 10.43 (1H, s, N-H), 7.66 (2H, d, J = 8.5 Hz, Ar-H), 7.60 (1H, d, J = 6 Hz, Ar-H), 7.48 (1H, d, J = 8 Hz, Ar-H), 7.40 (1H, t, J = 9 Hz, Ar-H), 7.31 (2H, d, J = 8 Hz, Ar-H), 2.73 (3H, s, -CH3). ^13^C NMR (101 MHz, DMSO_6_) δ: 170.72, 161.63, 159.63, 159.48, 155.27, 144.51, 138.21, 134.19, 133.20, 133.12, 126.19, 122.11, 121.77, 115.41, 115.24, 114.99, 13.00. HRMS (ESI): *m*/*z* calcd. for C_18_H_11_ClFN_2_O_3_ [M + H]^+^: 415.0120; found: 515.0125.

### 2.2. Streamlined DNA Preparation and AMPA Receptor Transfection in HEK293 Cells

#### 2.2.1. DNA Preparation

Using the QIAGEN Plasmid Mini Kit (catalog No.12123, QIAGEN, Hilden, Germany), a single colony from a streaked selective plate was picked and then inoculated into an LB medium supplemented with the appropriate selective antibiotic priming for incubation at 37 °C for 8 h. This initial culture was diluted into 3 mL of a selective LB medium and allowed to grow under incubation for 12–16 h at 37 °C to ensure enough bacterial mass. Bacterial cells were collected through centrifugation, and the resulting pellet was reconstituted to ensure homogeneity. Lysis was commenced by adding 0.3 mL of Buffer P2, and the entire mixture was gently inverted to mix thoroughly 4–6 times. The lysate was centrifuged to separate cellular debris from the plasmid-containing supernatant. A QIAGEN-tip 20 column with 1 mL of Buffer QBT was equilibrated to purify. This supernatant was applied to the column, allowing gravity flow for plasmid DNA binding to the resin. The column was then washed with Buffer QC to eliminate impurities; the elution of plasmid DNA was performed using 0.8 mL of Buffer QF. For DNA precipitation, isopropanol was added to the eluate, mixed, and centrifuged to form a DNA pellet. The pellet was washed with ethanol, centrifuged, and air-dried. Finally, the DNA was resuspended in an appropriate volume of the buffer. The concentration and purity of plasmid DNA were also determined via spectrophotometry at 260 nm. A260 readings from 0.1 to 1.0 guaranteed accuracy. The integrity of the DNA was also checked using agarose gel electrophoresis.

#### 2.2.2. Human Embryonic Kidney Cell Preparation

HEK293T cells (catalog number 85120602, Sigma-Aldrich, Darmstadt, Germany) were maintained in Dulbecco’s Modified Eagle Medium (DMEM; Sigma-Aldrich, Darmstadt, Germany), supplemented with 10% fetal bovine serum (FBS; Gibco, Thermo Fisher Scientific, Waltham, MA, USA), 0.1 mg/mL streptomycin (Biological Industries, Beit-Haemek, Israel), and 1 mM sodium pyruvate (Sigma-Aldrich, Darmstadt, Germany) at 37 °C with 5% CO_2_. Cells were passed every other week and maintained until passage 20 for further experimentation. Transfection was performed using a jetPRIME^®^ transfection reagent (Catalog No: 114-07, Polyplus, New York, NY, USA) according to the manufacturer’s protocol.

For transfection, 0.5 µg DNA, 0.5 µg of EGFP, and 1 µg of PRK-5 Vector Plasmid were diluted in 200 µL jetPRIME buffer. The mixture was vortexed briefly and centrifuged, followed by the addition of 4 µL jetPRIME, vortexing for 10 s, and brief centrifugation. The mixture was incubated for 10 min at room temperature. The cell culture medium was replaced with 2 mL fresh DMEM (not poured directly onto the cells but down the side of the dish). A total of 200 µL of the transfection mix was added dropwise per well and evenly distributed. The plate was gently tilted to ensure uniform distribution, and cells were incubated for 24 h before analysis.

Following transfection, cells were transferred to laminin-coated coverslips (1 mg/mL; Sigma-Aldrich, Darmstadt, Germany), providing a suitable surface for electrophysiology and imaging applications. Cells were maintained under standard culture conditions and analyzed post-transfection.

#### 2.2.3. Transient cDNA Transfection

The AMPA receptor subunits utilized in this study were of the flip isoform. The flip variant of the GluA2 subunit was originally provided by S. F. Heinemann (Salk Institute, La Jolla, CA, USA) in a pBlueScript plasmid. This plasmid was subsequently sub-cloned into a pRK vector to enable expression in HEK293 cells, which were sourced from Sigma, Germany. Additionally, the unedited version of GluA2 (R607Qjj, flip isoform) and enhanced green fluorescent protein (EGFP), both contained in pRK5 vectors, were generously supplied by P. H. Seeburg (Max Planck Institute for Medical Research, Heidelberg, Germany). Chemical-mediated ways induced transfection. GluA2 homomers were expressed into HEK293 cells by the co-transfection of 1 μg GluA2 plasmid with an equal amount of GFP expression vectors (1:1 ratio) into the cells. In addition, co-transfection was conducted for heteromeric GluA2/3 subunits at a ratio of 1:1.2 for optimum expression. For post-transfection experiments, cells were plated in 10% fetal calf serum and antibiotics in DMEM in Petri plates. These cultures were maintained under carefully controlled conditions—37 °C and 5% CO_2_ within a humidified incubator. The selection of cells for recordings was based on fluorescence intensity. Highly GFP-positive cells were identified and isolated for further analysis, ensuring precise targeting of transfected populations [45].

### 2.3. Electrophysiology Recordings

#### 2.3.1. Experimental Setup

Whole-cell patch clamp recordings were performed on HEK293t cells 36–48 h after transfection. Experiments were conducted at 22 °C, maintaining a holding potential of −60 mV, using an Integrated Patch Amplifier (IPA) from Sutter Instruments (Novato, CA, USA). Data acquisition was managed with SutterPatch Software v. 1.1.1 (Sutter Instruments), employing a sampling rate of 10 kHz and setting the low-pass filter at 2 kHz. Patch electrodes, crafted from borosilicate glass, had a resistance range of 2–4 MΩ. The external solution contained 150 mM NaCl, 2.8 mM KCl, 0.5 mM MgCl_2_, 2 mM CaCl_2_, and 10 mM HEPES, adjusted to a pH of 7.4 using NaOH. The internal pipette solution was composed of 110 mM CsF, 30 mM CsCl, 4 mM NaCl, 0.5 mM CaCl_2_, 10 mM trypsin-EDTA solution B (0.25%), 0.05% EDTA, and 10 mM HEPES, with the pH adjusted to 7.2 using CsOH.

A dual-barrel glass pipette (theta tube) was employed for the rapid delivery of glutamate and other solutions. The theta tube was attached to a high-speed piezoelectric solution exchange system (Automate Scientific, Berkeley, CA, USA). To assess the efficiency of solution exchange, open-tip potentials were measured by applying solutions with different ionic compositions after the patch was expelled from the pipette. The time required for a 10–90% change in the solution was generally less than 500 ms.

Data analysis was performed with Igor Pro8.04 software (Wave Metrics, Inc., Portland, OR, USA). AMPA receptor deactivation and desensitization kinetics were measured by applying 10 mM glutamate for 1 ms and 500 ms, respectively. The currents from the AMPA receptor were fitted to a double-exponential model to quantify deactivation and desensitization time constants measured from the double-exponential fits. The weighted tau (τ_w_) is calculated as: τ_w_ = (τf × af) + (τs × as), where τf and τs are the time constants of fast and slow exponential components and af and as represent their relative amplitudes [46].

#### 2.3.2. Statistical Analysis

Data are presented as the mean ± standard deviation (SD). The total number of HEK293 cells engaged was n = 10. The experimental groups were compared to the wild type using a one-way analysis of variance (ANOVA). Statistical significance was assessed using *p* values: *p* < 0.05 (*) indicated significance, *p* < 0.01 (**) indicated high significance, and *p* < 0.001 (***) indicated very high significance. The value “ns” indicates non-significance. Concentration–response relationships were modeled using the Hill equation with the resulting combined plots via GraphPad Prism software (version 6.01, GraphPad Software). The data presented are the means from 3–4 experimental replicates.

## 3. Results

### 3.1. Chemistry

**Scheme 1:**
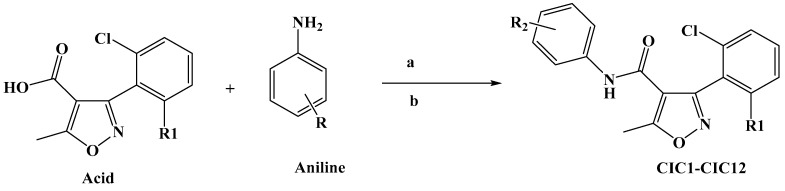
Synthesis of isoxazole–carboxamide derivatives (CIC1–CIC12). (a): EDC (1.3 equiv), (b): DMAP (0.3 equiv), DCM (15 mL), under inert gas, stirring for 48 h.

**Figure 1:**
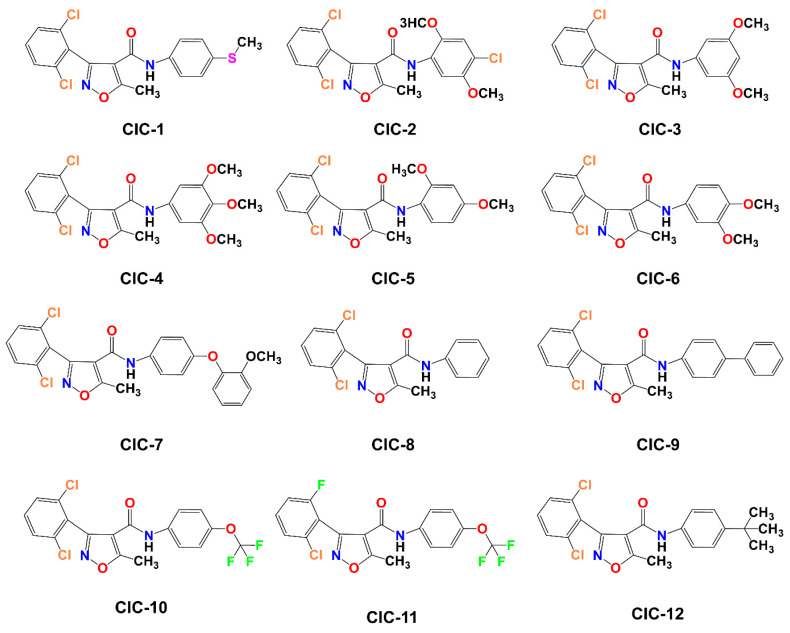
Illustration of the molecular structures of 12 Isoxazole-4-carboxamide derivatives, labeled CIC-1 through CIC-12.

Scheme 1 illustrates the approach adopted for preparing the compound CIC-1-12. The final isoxazole–carboxamide derivatives were synthesized through a coupling process facilitated by DMAP, serving as a nucleophilic catalyst, and EDCI, acting as a coupling reagent. After initiating the reaction and allowing it to proceed for 30 min, an aniline derivative was introduced to complete the transformation [47]. The coupling reaction’s mechanism employing EDC as the activating agent was outlined before [44,48]. The final CIC compounds are: *3-(2,6-dichlorophenyl)-5-methyl-N-(4-(methylthio)phenyl)isoxazole-4-carboxamide (CIC-1), N-(4-chloro-2,5-dimethoxyphenyl)-3-(2,6-dichlorophenyl)-5-methylisoxazole-4-carboxamide (CIC-2), 3-(2,6-dichlorophenyl)-N-(3,5-dimethoxyphenyl)-5-methylisoxazole-4-carboxamide (CIC-3), 3-(2,6-dichlorophenyl)-5-methyl-N-(3,4,5-trimethoxyphenyl)isoxazole-4-carboxamide (CIC-4), 3-(2,6-dichlorophenyl)-N-(2,4-dimethoxyphenyl)-5-methylisoxazole-4-carboxamide (CIC-5), 3-(2,6-dichlorophenyl)-N-(3,4-dimethoxyphenyl)-5-methylisoxazole-4-carboxamide (CIC-6), 3-(2,6-dichlorophenyl)-N-(4-(2-methoxyphenoxy)phenyl)-5-methylisoxazole-4-carboxamide (CIC-7), 3-(2,6-dichlorophenyl)-5-methyl-N-phenylisoxazole-4-carboxamide (CIC-8), N-([1,1′-biphenyl]-4-yl)-3-(2,6-dichlorophenyl)-5-methylisoxazole-4-carboxamide (CIC-9), 3-(2,6-dichlorophenyl)-5-methyl-N-(4-(trifluoromethoxy)phenyl)isoxazole-4-carboxamide (CIC-10), 3-(2-chloro-6-fluorophenyl)-5-methyl-N-(4-(trifluoromethoxy)phenyl)isoxazole-4-carboxamide (CIC-11),* and *N-(4-(tert-butyl)phenyl)-3-(2,6-dichlorophenyl)-5-methylisoxazole-4-carboxamide (CIC-12)* (Figure 1).

### 3.2. Inhibitory Effects of Isoxazole-4-Carboxamide Derivatives on the AMPA Receptor

To investigate the inhibitory effects of isoxazole-4-carboxamide derivatives on AMPA receptor activity, we measured peak current amplitudes in HEK293t cells expressing either GluA2 or GluA2/3 receptors using whole-cell patch clamp electrophysiology. Application of 10 mM AMPA induced inward currents in both cell lines, representing the baseline AMPA receptor activity. Subsequent co-application of each of the 12 CIC derivatives at a concentration of 16 µM allowed us to assess their inhibitory effects on these AMPA-evoked currents.

**Figure 2:**
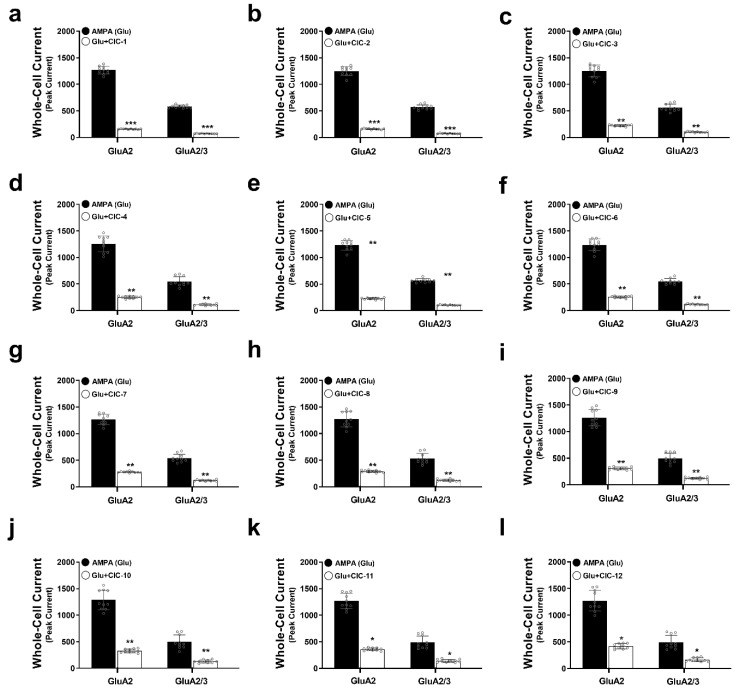
Effects of Isoxazole-4-carboxamide derivatives on AMPA receptor-mediated whole-cell currents. This figure demonstrates the inhibitory actions of Isoxazole-4-carboxamide derivatives on the whole-cell currents mediated by GluA2 and GluA2/3 AMPA receptor subtypes. Panels (**a**–**l**) represent data for the individual CIC compounds. Whole-cell patch clamp recordings were carried out on HEK293t cells expressing GluA2 or GluA2/3 subunits. AMPA-induced currents (black bars) are then compared to currents recorded in the presence of 16 µM CIC compounds (white bars). One-way ANOVA statistical tests were performed, * *p* < 0.05, ** *p* < 0.01, and *** *p* < 0.001, reflecting a significant reduction in currents. All the recordings were performed in whole-cell patch clamp mode at −60 mV, pH 7.4, and 22 °C. Data thus are shown as means ± SD.

Our results demonstrated that all tested CIC derivatives significantly attenuated peak AMPA-induced currents in GluA2 and GluA2/3 receptors (Figure 2a–l, Tables S1–S12, Figure S1). However, the degree of inhibition exhibited considerable variability among the compounds. Notably, CIC-1 and CIC-2 exerted the most potent inhibitory effects. CIC-1 reduced the peak current amplitude by approximately 8-fold in both GluA2 (Control: 1267 ± 75 pA, CIC-1: 156 ± 7 pA, *p* < 0.001) and GluA2/3 (Control: 580 ± 27 pA, CIC-1: 73 ± 5 pA, *p* < 0.001) receptors (Table S1, Figure 2a), indicating a substantial suppression of AMPA receptor-mediated currents. Similarly, CIC-2 diminished AMPA responses by approximately 7.8-fold in both GluA2 (Control: 1248 ± 86 pA, CIC-2: 160 ± 12 pA, *p* < 0.001) and GluA2/3 (Control: 569 ± 44 pA, CIC-2: 75 ± 6 pA, *p* < 0.001) receptors (Table S2, Figure 2b). The inhibitory effects of the remaining compounds (CIC-3 through CIC-12) were less pronounced, with varying degrees of current reduction detailed in Tables S3–S12 and Figure 2c–l.

**Figure 3:**
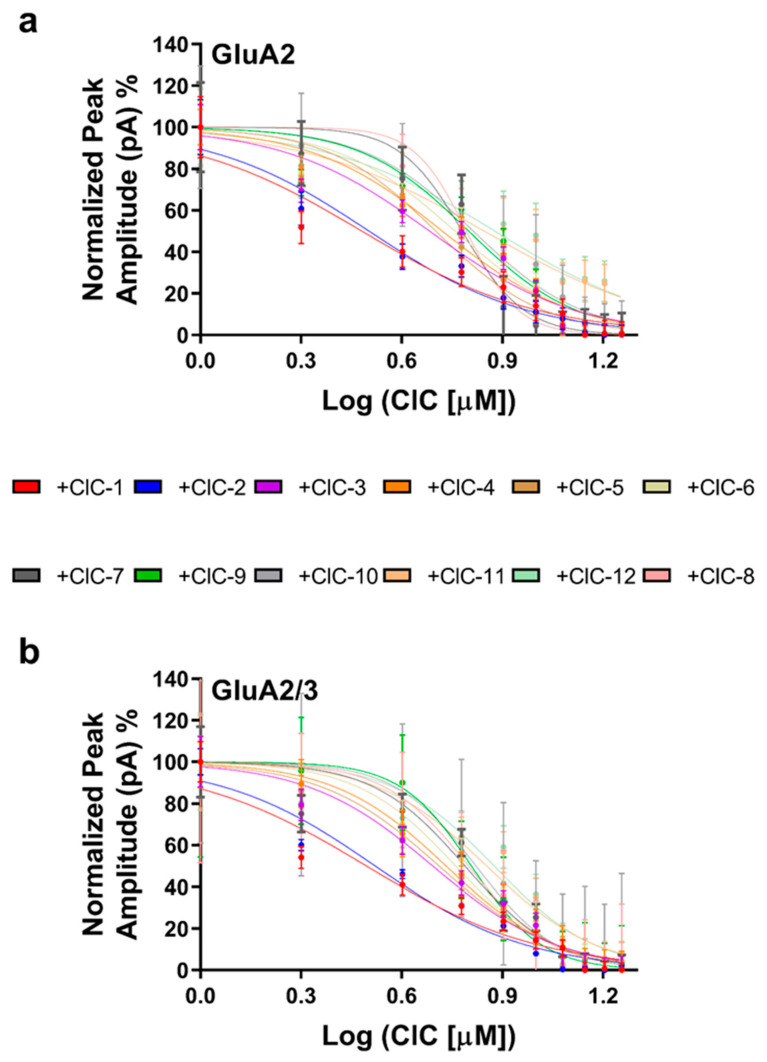
Dose-dependent inhibition of AMPAR subunits by CIC compounds. Whole-cell peak currents were recorded at −60 mV in HEK293t cells to evaluate the inhibitory effects caused by the various CIC compounds on the AMPA receptor subunits. The results regarding dose–response curves are shown as a percentage of the control current amplitude. For the assay, the concentration of CIC compounds was 16 μM. IC_50_ values were determined with non-linear regression in GraphPad Prism. Recordings were performed at pH 7.4 and 22 °C. The inhibitory effect on GluA2 receptors is shown in (**a**), and the dose–response relationship for the GluA2/3 receptors in (**b**) shows differences in inhibitory potency by the CIC compounds. Data points reflect results from more than one experiment, and error bars indicate ± SD for 10 cells.

To quantify the inhibitory potency of each derivative, concentration–response curves were constructed (Figure 3a,b), and IC_50_ values were determined (Table S13). This analysis confirmed the potency of CIC-1 and CIC-2, which exhibited IC_50_ values in the low micromolar range (3.03 µM and 3.12 µM for GluA2, and 3.12 µM and 3.32 µM for GluA2/3, respectively). The other derivatives displayed higher IC_50_ values, ranging from 4.82 µM to 7.54 µM for GluA2 and 4.92 µM to 7.50 µM for GluA2/3.

**Figure 4:**
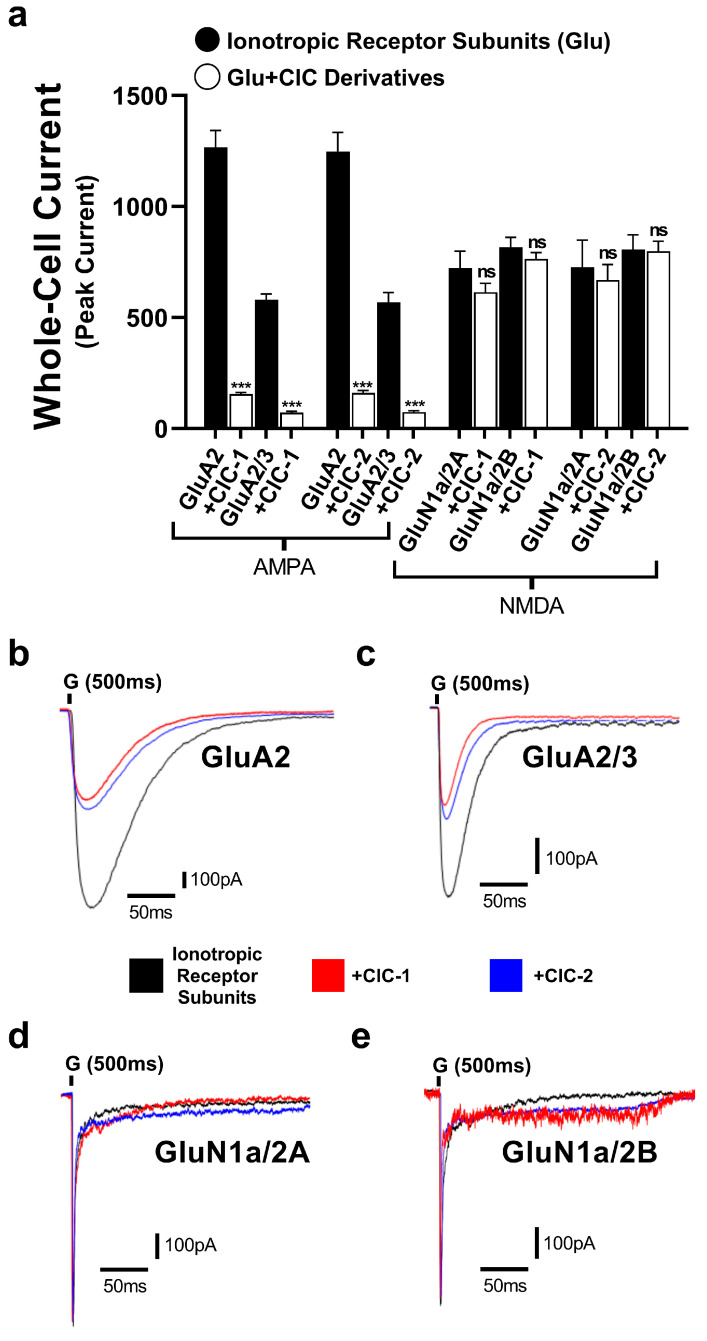
The inhibitory actions of Isoxazole-4-carboxamide derivatives on the whole-cell currents mediated by GluA2, GluA2/3 AMPA receptor subtypes and GluN1a/2A, GluN1a/2A NMDA receptor subtypes. (**a**) represents data for CIC-1 and CIC-2 compounds. AMPA- and NMDA-induced currents (black bars) are then compared to currents recorded in the presence of 16 µM CIC compounds (white bars). Panels (**b**–**e**) show representative traces of whole-cell current responses to a 1 ms administration of 10 mM glutamate for the subunits. The traces show normalized responses for glutamate alone (black) and glutamate in combination with CIC compounds (color-coded). The recording was performed in HEK293t cells at −60 mV, pH 7.4, and 22 °C. Data present mean ± SD from 3–4 different experiments. *** *p* < 0.001; and “ns” indicates not significant.

To evaluate the selectivity of CIC-1 and CIC-2 derivatives, their effects on GluN1a/2A and GluN1a/2B of the NMDA receptors were tested. The normalized peak current responses of the subunits alone and with CIC-1 and CIC-2 were determined (Figure 4). The results showed CIC-1 and CIC-2 having only mild effects in the normalized peak current of GluN1a/2A and GluN1a/2B, which were considered statistically not significant. These results suggest CIC-1 and CIC-2 do not modulate the subunits of the NMDA receptors.

### 3.3. Effects of Isoxazole-4-Carboxamide Derivatives on AMPA Receptor Deactivation and Desensitization Kinetics

**Figure 5:**
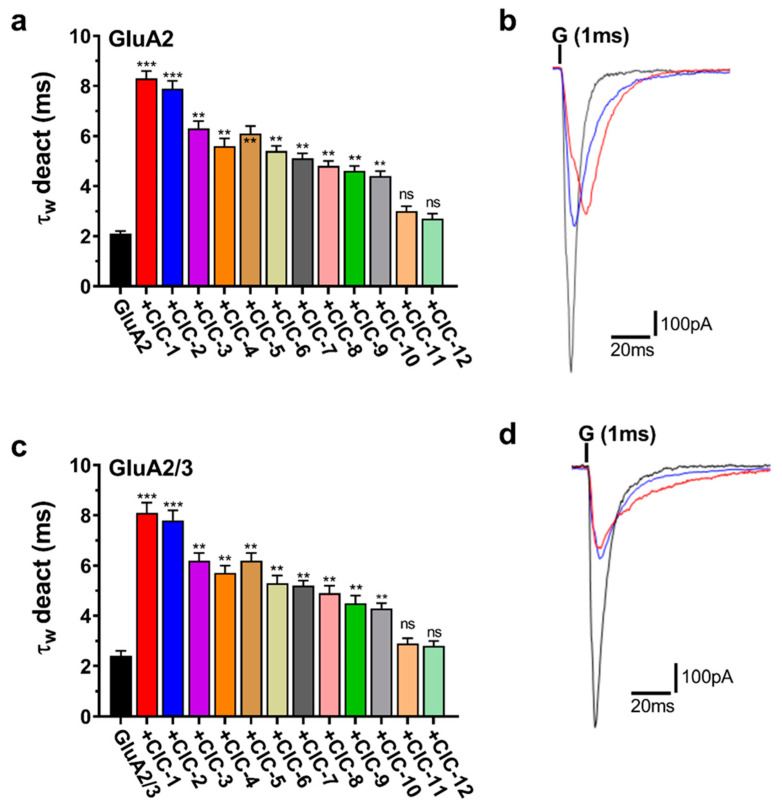
Deactivation kinetics (τ_w_ deact) of AMPAR subunits in the presence of CIC compounds. Panels (**a**,**c**) show τ_w_ deact weighted time constants for both GluA2 and GluA2/3 receptors activated by CIC compounds. Bar graphs show that CIC compounds extend the duration of deactivation kinetics. Statistical analysis was performed with the control condition (receptors exposed to glutamate alone) for each. Increases in τ_w_ deact that reached significance are marked with ** *p* < 0.01, *** *p* < 0.001, and “ns” for not significant (one-way ANOVA). Panels (**b**,**d**) show representative traces of whole-cell current responses to a 1 ms administration of 10 mM glutamate for GluA2 and GluA2/3, respectively. The traces show normalized responses for glutamate alone (black) and glutamate in combination with CIC compounds (CIC-1 in red, and CIC-2 in blue). The recording was performed in HEK293 cells at −60 mV, pH 7.4, and 22 °C. Data present mean ± SD from 3–4 different experiments.

Deactivation, the closing of the AMPA receptor channel after removing glutamate, was significantly affected by the CIC derivatives (Figure 5a,c). Most notably, CIC-1 and CIC-2 profoundly slowed deactivation, increasing the deactivation time constant (τ_deac_) by approximately 4-fold in both GluA2 and GluA2/3 receptors. In GluA2 receptors, τ_deac_ was prolonged from a control value of 2.1 ± 0.1 ms to 8.3 ± 0.3 ms upon CIC-1 application (*p* < 0.001). Similarly, in GluA2/3 receptors, τ_deac_ increased from 2.4 ± 0.2 ms in control conditions to 8.1 ± 0.4 ms with CIC-1 (*p* < 0.001) (Table S1, Figure 5a,c). CIC-2 exhibited comparable effects, quadrupling τ_deac_ in GluA2 and GluA2/3 receptors (Table S2).

Beyond CIC-1 and CIC-2, several other derivatives also significantly impacted deactivation kinetics, however, to a lesser extent. CIC-3, CIC-4, CIC-5, CIC-6, CIC-7, CIC-8, CIC-9, and CIC-10 all demonstrated a statistically significant prolongation of τ_deac_ in both GluA2 and GluA2/3 receptors (*p* < 0.05, *p* < 0.01, respectively) (Figure 5a,c, Tables S3–S9). The magnitude of this effect varied among these compounds, with τ_deac_ values ranging from approximately 3.5 ms to 6 ms in GluA2 and 4 ms to 7 ms in GluA2/3 receptors. In contrast, CIC-11 and CIC-12 did not elicit statistically significant changes in deactivation kinetics in either GluA2 or GluA2/3 receptors (Figure 5a,c, Tables S10–S12).

Representative current traces illustrating the effects of CIC-1, CIC-2, and CIC-3 on deactivation are presented in Figure 5b,d. These traces demonstrate the prolonged current decay in the presence of CIC-1 and CIC-2, consistent with their significant impact on τ_deac_.

**Figure 6:**
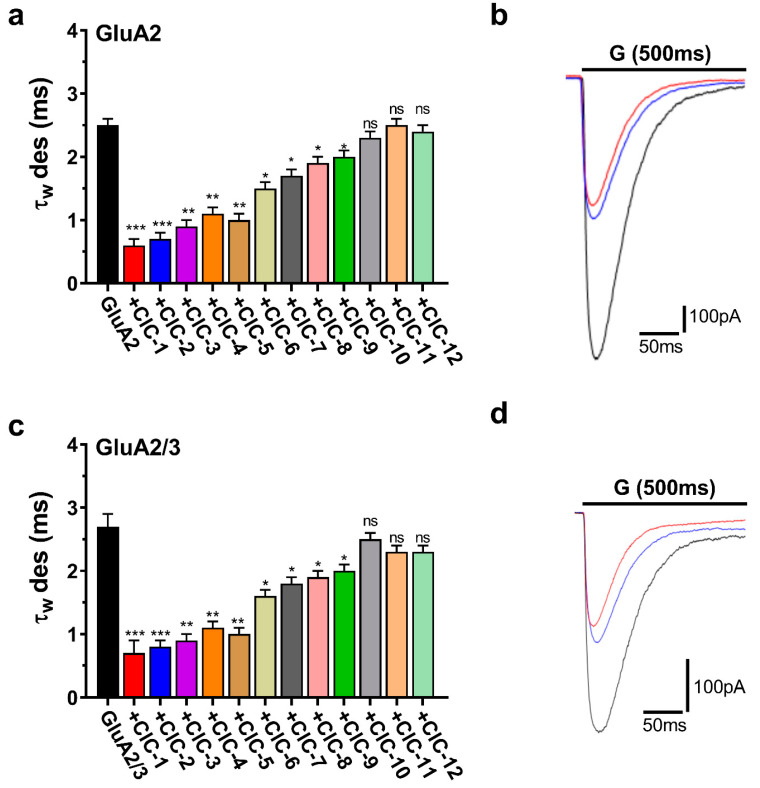
Desensitization kinetics (τ_w_ des) of AMPAR subunits in the presence of CIC compounds. The plots are normalized whole-cell current traces (pA) for AMPAR subunits subjected to 500 ms applications of 10 mM glutamate (“G” above the traces) in HEK293 cells expressing homomeric GluA2 (**a**,**b**) and heteromeric GluA2/3 (**c**,**d**). Traces include responses to glutamate alone (black) or glutamate with combinations of CIC compounds represented by red for CIC-1 and blue for CIC-2. The bar graphs (**a**,**c**) illustrate the weighted desensitization time constants (τ_w_ des) for GluA2 and GluA2/3, demonstrating the impact of CIC compounds on controlled desensitization kinetics. Differences regarding desensitization rates significant from the control are indicated by * *p* > 0.05, ** *p* < 0.01, *** *p* < 0.001, and “ns” denotes no significant change (one-way ANOVA). All recordings came under a whole-cell patch clamp condition at −60 mV, pH 7.4, and 22 °C. Data are displayed as mean ± SD from 3–4 different experiments.

CIC-1 and CIC-2 accelerated desensitization, representing the rapid current amplitude decline during sustained glutamate application. CIC-1 markedly reduced the desensitization time constant (τ_des_) in both GluA2 and GluA2/3 receptors. In GluA2 receptors, τ_des_ decreased from a control value of 2.5 ± 0.1 ms to 0.6 ± 0.1 ms upon CIC-1 application (*p* < 0.001). Similarly, in GluA2/3 receptors, τ_des_ was reduced from 2.7 ± 0.2 ms in control conditions to 0.7 ± 0.2 ms with CIC-1 (*p* < 0.001) (Table S1, Figure 6a,c). CIC-2 exerted similar effects on τ_des_, accelerating desensitization in GluA2 and GluA2/3 receptors (Table S2, Figure 6a,c).

The remaining CIC derivatives exhibited more varied effects on desensitization kinetics. CIC-3 significantly accelerated desensitization in both GluA2 and GluA2/3 receptors, decreasing τ_des_ to a similar extent as CIC-1 and CIC-2 (Tables S1–S3). CIC-4, CIC-5, CIC-6, CIC-7, CIC-8, and CIC-9 also accelerated desensitization, although to a lesser degree, with statistically significant reductions in τ_des_ observed in both receptor subtypes (*p* < 0.05, *p* < 0.01) (Tables S4–S6, Figure 6a,c). Finally, CIC-10, CIC-11, and CIC-12 did not significantly alter desensitization kinetics (Tables S10–S12, Figure 6a,c).

Representative current traces illustrating the effects of CIC-1, CIC-2, and CIC-3 on desensitization are displayed in Figure 6b,d. These traces visually demonstrate the faster decay of the current during sustained glutamate application in the presence of these compounds, consistent with their acceleration of τ_des_.

## 4. Discussion

The therapeutic potential of isoxazole-4-carboxamide derivatives in chronic inflammatory pain management through AMPA receptor regulation represents a significant advancement in the search for non-opioid analgesics. Our electrophysiological studies have elucidated the modulatory effects of these derivatives on AMPA receptors, which are integral to nociceptive transmission and the pathophysiology of chronic pain. The results demonstrated that isoxazole-4-carboxamide derivatives, particularly CIC-1 and CIC-2, exhibited potent inhibitory effects on AMPA receptor activity. The 8-fold and 7.8-fold reductions in peak current amplitudes for CIC-1 and CIC-2 indicate a strong capacity to suppress AMPA receptor-mediated currents in both GluA2 and GluA2/3 receptor subtypes. This inhibition is particularly relevant given the role of AMPA receptors in mediating excitatory neurotransmission in the spinal dorsal horn, where their overactivity is associated with heightened pain sensitivity and the development of chronic pain states [49,50]. The low IC_50_ values for these compounds further underscore their potential as effective therapeutic agents, as they can inhibit receptor activity at relatively low concentrations.

The similar effects observed on GluA2 and GluA2/3 receptors suggest that our compounds target conserved regulatory mechanisms. However, the slightly higher potency observed for GluA2 homomers (IC_50_ 3.03 μM vs. 3.12 μM for GluA2/3) might indicate subtle subtype preferences that could be exploited in future drug development. Recent structural studies have identified subunit-specific regulatory sites that could explain these differences [51,52].

This unique combination of effects on channel kinetics might be explained by considering the structural dynamics of AMPA receptors. Recent cryo-EM studies have revealed distinct conformational states associated with channel opening, closing, and desensitization [53,54]. The apparent paradox of prolonged deactivation coupled with accelerated desensitization suggests our compounds might preferentially stabilize certain conformational intermediates while destabilizing others. The prolonged deactivation (τ_deac_ increased ~4-fold) suggests stabilization of a pre-open or alternative closed state that persists after glutamate unbinding, while the accelerated desensitization (τ_des_ decreased by ~75%) indicates facilitated transition to desensitized states during sustained glutamate exposure. By prolonging activation after brief glutamate exposure, these compounds might promote a refractory period that reduces the temporal summation of synaptic inputs. This mechanism would be particularly relevant in conditions of high-frequency synaptic activation, which often occurs in pathological pain states [55].

Through multiple mechanisms, this kinetic profile could be particularly beneficial in pathological pain states. Sustained AMPA receptor activation contributes significantly to central sensitization, a key mechanism driving chronic pain states, through increased neuronal excitability, synaptic plasticity, and neuroinflammation [56,57,58]. The prolonged deactivation observed with our compounds could effectively limit the duration of AMPA receptor activation following brief glutamate release, modulating AMPAR-mediated Na+ currents. This diminishes postsynaptic depolarization and hinders action potential generation, mitigating the development of central sensitization. Furthermore, the accelerated desensitization may prevent the temporal summation of nociceptive inputs, a critical factor in wind-up and the maintenance of chronic pain [56]. This dual modulation of AMPA receptor kinetics represents a novel mechanism for pain relief, potentially offering advantages over existing therapies that primarily focus on simple receptor blockade.

Our findings also have broader implications for understanding AMPA receptor pharmacology. The ability of these compounds to simultaneously modify multiple aspects of the channel function suggests they might interact with regions of the receptor involved in conformational coupling between these processes. This observation aligns with recent structural studies showing that AMPA receptor gating involves complex allosteric interactions between domains [59]. The efficacy gradient observed across isoxazole-4-carboxamide derivatives (CIC-1 through CIC-12) provides valuable insights into potential interaction mechanisms. The notably higher potency of CIC-1 and CIC-2 than the other derivatives suggests specific molecular features that enhance their interaction with AMPA receptors. Recent studies have identified several allosteric binding sites on AMPA receptors that could accommodate small molecule modulators [60,61]. The differential effects of our compounds might reflect varying abilities to access or stabilize these sites.

The 2,6-dichlorophenyl moiety, common to all compounds, enhances binding primarily through hydrophobic interactions with residues within the AMPA receptor binding pocket (e.g., PHE623, LEU620) [62]. The electron-withdrawing nature of chlorine further optimizes this interaction, likely through π–π stacking and halogen bonding. This core structure is particularly effective in CIC-1 and CIC-2 due to additional favorable substituents. In CIC-1, the 4-(methylthio)phenyl group significantly boosts lipophilicity and enhances binding via sulfur-mediated hydrophobic contacts. Methoxy groups (-OCH_3_), present in CIC-2 through CIC-7, significantly enhance activity through hydrogen bonding and resonance effects, stabilizing receptor interactions. CIC-2, with its 4-chloro-2,5-dimethoxyphenyl substitution, showcases this most effectively, maximizing π–π stacking and hydrogen bonding, resulting in superior activity. The 3,4,5-trimethoxyphenyl moiety in CIC-4 provides multiple interaction points, further enhancing receptor affinity, although its slightly lower potency than CIC-2 suggests steric factors play a role.

The spatial arrangement between the phenyl and isoxazole rings is critical for effective binding. CIC-1 and CIC-2 demonstrate near-ideal non-coplanarity, aligning effectively with the receptor’s binding pocket. In contrast, diminished activity in CIC-11 (2-chloro-6-fluorophenyl) and CIC-12 (tert-butyl group) underscores the impact of steric hindrance. These bulky substituents likely interfere with optimal interactions at the binding site, aligning with recent structural data regarding AMPA receptor allosteric sites [50]. The unsubstituted phenyl ring in CIC-8 exhibits reduced activity, attributed to the lack of hydrogen bonding or π–π stacking interactions, apart from those provided by the 2,6-dichlorophenyl moiety. The biphenyl substitution in CIC-9 also shows slightly diminished activity, likely due to the increased steric bulk influencing receptor interactions, despite the potential for increased hydrophobic interactions. While exhibiting moderate activity, the trifluoromethoxy group (-OCF_3_) in CIC-10 suffers from steric hindrance that offsets the electron-withdrawing effect.

This analysis underscores the importance of precise molecular interactions for effective AMPA receptor modulation. The observed sensitivity to steric hindrance and the requirement for optimal spatial arrangements of substituents highlight the complexity of allosteric modulation at AMPA receptors. This intricate interplay between the structure and function is particularly relevant considering the multifaceted role of AMPA receptors in chronic pain, where their activity is intricately linked to inflammatory processes. The recent literature has highlighted the role of inflammatory cytokines in enhancing AMPA receptor activity, thereby increasing spinal neuron excitability and pain sensitivity [63]. Additionally, sustained AMPA receptor activation contributes to various pathological processes in chronic pain conditions, including neuroinflammation and maladaptive synaptic plasticity [57]. This suggests that targeting AMPA receptors could provide a novel therapeutic strategy for chronic inflammatory pain management [64]. Modulating receptor kinetics and reducing overall current amplitude, as well as isoxazole-4-carboxamide derivatives, might help normalize these downstream processes.

While our in vitro findings demonstrate potent AMPA receptor modulation by the CIC derivatives, in vivo confirmation is needed to establish the analgesic efficacy of the compounds. The established role of AMPA receptors in pain pathways, particularly within the spinal dorsal horn, suggests a potential mechanism by which these compounds could contribute to pain relief. By inhibiting AMPA receptor activity, these derivatives may reduce the excitability of pain-transmitting neurons, potentially attenuating pain signaling. The observed selectivity of the CIC derivatives for AMPA receptors over NMDA receptors further strengthens their potential as therapeutic agents. This selectivity suggests a reduced likelihood of off-target effects on other glutamate receptors, potentially minimizing side effects commonly associated with non-selective glutamate receptor inhibitors.

Future directions should include detailed structural studies to elucidate the precise binding mode of these compounds, possibly using cryo-EM or X-ray crystallography combined with molecular dynamics simulations. Additionally, investigating these compounds in animal models of chronic pain, particularly their effects on synaptic plasticity and neuroinflammation in vivo, would help validate their therapeutic potential. Recent advances in real-time imaging of synaptic function in awake animals could provide valuable insights into how these compounds modify pain circuit dynamics [65].

## 5. Conclusions

This research studied the inhibitory impact of previously published isoxazole-4-carboxamide derivatives (CIC-1 to CIC-12) on AMPA receptor activation and their potential effectiveness in chronic pain management. Using whole-cell patch clamp electrophysiology, we determined that CIC-1 and CIC-2 are the most effective inhibitors, markedly reducing peak current amplitudes and modifying receptor kinetics by prolonging deactivation and accelerating desensitization. These compounds have been associated with low IC50 values and have a promising dual mechanism of action, which may reduce the central sensitization associated with chronic pain. Other derivatives tested showed only moderate potency, underlining that only certain substituents are important for a remarkable increase in potencies. Across all assays, CIC-1 and CIC-2 consistently demonstrated potent modulation of AMPA receptors, suggesting their potential for further in vivo investigation as non-opioid analgesics targeting this receptor subtype.

## Data Availability

The original contributions presented in this study are included in the article/supplementary material. Further inquiries can be directed to the corresponding author.

## Supplementary Materials

The following supporting information can be downloaded at: https://www.mdpi.com/article/10.3390/jox15020040/s1, Figure S1: Inhibitory Effect of CIC Derivatives on Glutamate-Induced Currents; Table S1–S12: Whole-cell recordings of data analysis for CIC compounds; Table S13. IC50 calculated values for CIC compounds; Spectral data of Isoxazole-Carboxamide derivatives.
