# Supplementary material for: Regulating AMPA Receptors with Isoxazole-4-Carboxamide Derivatives: An Electrophysiological Study
Source: J Xenobiot. 2025 Mar 8;15(2):40. doi: 10.3390/jox15020040 (PMC11932207; doi:10.3390/jox15020040)
Supplement: Supplementary file 1 [file jox-15-00040-s001.zip › jox-3485237-supplementary.pdf]

Table of Contents

Table S1–S12. Whole-cell recordings of data analysis for CIC compounds …………………..3-10
Supplemental Table S13. IC_{50} calculated values for CIC compounds …….………..………..11
Figure S1. Inhibitory Effect of CIC Derivatives on Glutamate-Induced Currents….....……......12
Spectral data of Isoxazole-Carboxamide derivatives…………………………………...…....13

The data shown is the mean ± SD, with n = 10 being the number of patch cells in the whole-cell arrangement. A one-way ANOVA test was used to determine data significance, with thresholds of * $p < 0.05$, ** $p < 0.01$, *** $p < 0.001$, and ns (not significant).

Table S1. Whole-Cell Recordings for Compound CIC -1.

Receptor Name/Compounds abbreviation	GluA2 (Glutamate Alone)	ClC-1	Applying Glutamate Alone After ClC-1	n	A/A_I
**Amplitude (pA)**	1267±75	156±7***	1231±85	10	8.11±0.41
**t deact (ms)**	2.1±0.1	8.3±0.3***	N/R	10	N/R
**t des (ms)**	2.5±0.1	0.6±0.1***	N/R	10	N/R
**Receptor Name/Compounds abbreviation**	**GluA2/3 (Glutamate Alone)**	**ClC-1**	**Applying Glutamate Alone After ClC-1**	**n**	**A/A_I**
**Amplitude (pA)**	580±27	73±5***	562±28	10	8.02±0.49
**t deact (ms)**	2.4±0.2	8.1±0.4***	N/R	10	N/R
**t des (ms)**	2.7±0.2	0.7±0.2***	N/R	10	N/R

Table S2. Whole-Cell Recordings for Compound CIC -2.

Receptor Name/Compounds abbreviation	GluA2 (Glutamate Alone)	ClC-2	Applying Glutamate Alone After ClC-2	n	A/A_I
**Amplitude (pA)**	1248±86	160±12***	1213±94	10	7.79±0.42
**t deact (ms)**	N/R	7.9±0.3***	N/R	10	N/R
**t des (ms)**	N/R	0.7±0.1***	N/R	10	N/R
**Receptor Name/Compounds abbreviation**	**GluA2/3 (Glutamate Alone)**	**ClC-2**	**Applying Glutamate Alone After ClC-2**	**n**	**A/A_I**
**Amplitude (pA)**	569±44	75±6***	552±44	10	7.65±0.45
**t deact**	N/R	7.8±0.4***	N/R	10	N/R

(ms)					
t des (ms)	N/R	0.8±0.1***	N/R	10	N/R

Table S3. Whole-Cell Recordings for Compound CIC -3.

Receptor Name/Compounds abbreviation	GluA2 (Glutamate Alone)	ClC-3	Applying Glutamate Alone After ClC-3	n	A/A$_I$
Amplitude (pA)	1252±113	223±15**	1216±119	10	5.60±0.27
t deact (ms)	N/R	6.3±0.3**	N/R	10	N/R
t des (ms)	N/R	0.9±0.1**	N/R	10	N/R
**Receptor Name/Compounds abbreviation**	**GluA2/3 (Glutamate Alone)**	**ClC-3**	**Applying Glutamate Alone After ClC-3**	**n**	**A/A$_I$**
Amplitude (pA)	559±67	101±10**	542±66	10	5.50±0.26
t deact (ms)	N/R	6.2±0.3**	N/R	10	N/R
t des (ms)	N/R	0.9±0.1**	N/R	10	N/R

Table S4. Whole-Cell Recordings for Compound CIC -4.

Receptor Name/Compounds abbreviation	GluA2 (Glutamate Alone)	ClC-4	Applying Glutamate Alone After ISX-4	n	A/A$_I$
Amplitude (pA)	1256±153	250±22**	1220±157	10	5.0±0.23
t deact (ms)	N/R	5.6±0.3**	N/R	10	N/R
t des (ms)	N/R	1.1±0.1**	N/R	10	N/R
**Receptor Name/Compounds abbreviation**	**GluA2/3 (Glutamate Alone)**	**ClC-4**	**Applying Glutamate Alone After ClC-4**	**n**	**A/A$_I$**
Amplitude (pA)	548±92	110±16**	531±89	10	4.97±0.23
t deact (ms)	N/R	5.7±0.3**	N/R	10	N/R
t des (ms)	N/R	1.1±0.1**	N/R	10	N/R

Table S5. Whole-Cell Recordings for Compound ClC -5.

Receptor Name/Compounds abbreviation	GluA2 (Glutamate Alone)	ClC-5	Applying Glutamate Alone After ClC-5	n	A/A_I
Amplitude (pA)	1233±90	228±15**	1198±99	10	5.42±0.07
t deact (ms)	N/R	6.1±0.3**	N/R	10	N/R
t des (ms)	N/R	1.0±0.1**	N/R	10	N/R
**Receptor Name/Compounds abbreviation**	**GluA2/3 (Glutamate Alone)**	**ClC-5**	**Applying Glutamate Alone After ClC-5**	**n**	**A/A_I**
Amplitude (pA)	565±36	105±7**	548±40	10	5.37±016
t deact (ms)	N/R	6.2±0.3**	N/R	10	N/R
t des (ms)	N/R	1.0±0.1**	N/R	10	N/R

Table S6. Whole-Cell Recordings for Compound ClC -6.

Receptor Name/Compounds abbreviation	GluA2 (Glutamate Alone)	ClC-6	Applying Glutamate Alone After ClC-6	n	A/A_I
Amplitude (pA)	1237±112	256±19**	1202±120	10	4.84±0.23
t deact (ms)	N/R	5.4±0.2**	N/R	10	N/R
t des (ms)	N/R	1.5±0.1*	N/R	10	N/R
**Receptor Name/Compounds abbreviation**	**GluA2/3 (Glutamate Alone)**	**ClC-6**	**Applying Glutamate Alone After ClC-6**	**n**	**A/A_I**
Amplitude (pA)	550±51	116±10**	534±56	10	4.76±0.14
t deact (ms)	N/R	5.3±0.3**	N/R	10	N/R
t des (ms)	N/R	1.6±0.1*	N/R	10	N/R

Table S7. Whole-Cell Recordings for Compound CIC -7.

Receptor Name/Compounds abbreviation	GluA2 (Glutamate Alone)	ClC-7	Applying Glutamate Alone After ClC-7	n	A/A_I
Amplitude (pA)	1267±99	280±13**	1231±106	10	4.53±0.21
t deact (ms)	N/R	5.1±0.2**	N/R	10	N/R
t des (ms)	N/R	1.7±0.1*	N/R	10	N/R
**Receptor Name/Compounds abbreviation**	**GluA2/3 (Glutamate Alone)**	**ClC-7**	**Applying Glutamate Alone After ClC-7**	**n**	**A/A_I**
Amplitude (pA)	540±74	120±15**	524±76	10	4.49±0.21
t deact (ms)	N/R	5.2±0.2**	N/R	10	N/R
t des (ms)	N/R	1.8±0.1*	N/R	10	N/R

Table S8. Whole-Cell Recordings for Compound CIC -8.

Receptor Name/Compounds abbreviation	GluA2 (Glutamate Alone)	ClC-8	Applying Glutamate Alone After ClC-8	n	A/A_I
Amplitude (pA)	1271±147	291±22**	1235±151	10	4.36±0.19
t deact (ms)	N/R	4.8±0.2**	N/R	10	N/R
t des (ms)	N/R	1.9±0.1*	N/R	10	N/R
**Receptor Name/Compounds abbreviation**	**GluA2/3 (Glutamate Alone)**	**ClC-8**	**Applying Glutamate Alone After ClC-8**	**n**	**A/A_I**
Amplitude (pA)	529±98	123±20**	513±99	10	4.30±0.20
t deact (ms)	N/R	4.9±0.3**	N/R	10	N/R
t des (ms)	N/R	1.9±0.1*	N/R	10	N/R

Table S9. Whole-Cell Recordings for Compound CIC -9.

Receptor Name/Compounds abbreviation	GluA2 (Glutamate Alone)	ClC-9	Applying Glutamate Alone After ClC-9	n	A/AI
Amplitude (pA)	1262±156	280±13**	1226±158	10	4.10±0.18
t deact (ms)	N/R	4.6±0.2**	N/R	10	N/R
t des (ms)	N/R	2.0±0.1*	N/R	10	N/R
**Receptor Name/Compounds abbreviation**	**GluA2/3 (Glutamate Alone)**	**ClC-9**	**Applying Glutamate Alone After ClC-9**	**n**	**A/AI**
Amplitude (pA)	499±91	124±19**	484±89	10	4.01±0.19
t deact (ms)	N/R	4.5±0.3**	N/R	10	N/R
t des (ms)	N/R	2.0±0.1*	N/R	10	N/R

Table S10. Whole-Cell Recordings for Compound CIC -10.

Receptor Name/Compounds abbreviation	GluA2 (Glutamate Alone)	ClC-10	Applying Glutamate Alone After ClC-10	n	A/AI
Amplitude (pA)	1285±187	328±35**	1249±188	10	3.90±0.16
t deact (ms)	N/R	4.4±0.2**	N/R	10	N/R
t des (ms)	N/R	2.3±0.1ns	N/R	10	N/R
**Receptor Name/Compounds abbreviation**	**GluA2/3 (Glutamate Alone)**	**ClC-10**	**Applying Glutamate Alone After ClC-10**	**n**	**A/AI**
Amplitude (pA)	497±130	128±29**	482±128	10	3.86±0.18
t deact (ms)	N/R	4.3±0.2**	N/R	10	N/R
t des (ms)	N/R	2.5±0.1ns	N/R	10	N/R

Table S11. Whole-Cell Recordings for Compound CIC -11.

**Receptor Name/Compounds abbreviation**	**GluA2 (Glutamate Alone)**	**ClC-11**	**Applying Glutamate Alone After ClC-11**	**n**	**A/A$_I$**
**Amplitude (pA)**	1269±146	359±28*	1233±147	10	3.53±0.15
**t deact (ms)**	N/R	3.0±0.2^{ns}	N/R	10	N/R
**t des (ms)**	N/R	3.4	N/R	10	N/R
**Receptor Name/Compounds abbreviation**	**GluA2/3 (Glutamate Alone)**	**ClC-11**	**Applying Glutamate Alone After ClC-11**	**n**	**A/A$_I$**
**Amplitude (pA)**	491±116	141±29*	476±112	10	3.46±0.16
**t deact (ms)**	N/R	2.9±0.2^{ns}	N/R	10	N/R
**t des (ms)**	N/R	2.3±0.1^{ns}	N/R	10	N/R

Table S12. Whole-Cell Recordings for Compound CIC -12.

**Receptor Name/Compounds abbreviation**	**GluA2 (Glutamate Alone)**	**ClC-12**	**Applying Glutamate Alone After ClC-12**	**n**	**A/A$_I$**
**Amplitude (pA)**	1273±195	417±47*	1237±195	10	3.04±0.13
**t deact (ms)**	N/R	2.7±0.2^{ns}	N/R	10	N/R
**t des (ms)**	N/R	2.4±0.1^{ns}	N/R	10	N/R
**Receptor Name/Compounds abbreviation**	**GluA2/3 (Glutamate Alone)**	**ClC-12**	**Applying Glutamate Alone After ClC-12**	**n**	**A/A$_I$**
**Amplitude (pA)**	493±131	162±36*	478±127	10	3.01±0.14
**t deact (ms)**	N/R	2.8±0.2^{ns}	N/R	10	N/R
**t des (ms)**	N/R	2.3±0.1^{ns}	N/R	10	N/R

Table S13. IC_{50} values.

Receptor/Subunit	ClC Derivatives	ClC-1	ClC-2	ClC-3	ClC-4	ClC-5	ClC-6	ClC-7
**GluA2**	**IC_{50}**	3.03	3.18	4.82	5.26	5.03	5.74	6.03
	**Log IC_{50}**	0.48	0.50	0.68	0.72	0.70	0.76	0.78
	**R square**	0.93	0.96	0.93	0.94	0.98	0.94	0.97
		ClC-8	ClC-9	ClC-10	ClC-11	ClC-12		
		6.21	6.30	6.51	7.02	7.54		
		0.79	0.80	0.81	0.85	0.88		
		0.98	0.92	0.97	0.91	0.91		

Receptor/Subunit	ClC Derivatives	ClC-1	ClC-2	ClC-3	ClC-4	ClC-5	ClC-6	ClC-7
**GluA2/3**	**IC_{50}**	3.12	3.32	4.92	5.40	5.14	5.76	6.14
	**Log IC_{50}**	0.49	0.52	0.69	0.73	0.71	0.76	0.79
	**R square**	0.94	0.95	0.97	0.99	0.99	0.98	0.94
		ClC-8	ClC-9	ClC-10	ClC-11	ClC-12		
		6.30	6.42	6.62	7.13	7.50		
		0.80	0.81	0.82	0.85	0.88		
		0.98	0.99	0.97	0.93	0.92		

Supplemental Figure S1. Inhibitory Effect of CIC Derivatives on Glutamate-Induced Currents The bar graphs show the effects of CIC derivatives on the amplitude ratio (A/AI) of glutamate-induced currents through GluA2 (a) and GluA2/3 (b) AMPA receptor subunits. The amplitude ratio (A/AI) indicates the normalized response of currents upon exposure to specific CIC derivatives compared to the controlling condition (glutamate alone). Currents under specific applied CIC derivatives show greatly significant changes indicated by the following levels of significance: *p < 0.05, **p < 0.01, and ***p < 0.001 (one-way ANOVA). The heights of the bars indicate mean values ± SD. This shows that CIC derivatives are capable of inhibiting glutamate-induced AMPAR responses. Recordings were performed at -60 mV under whole-cell patch-clamp conditions, pH 7.4, and 22°C.

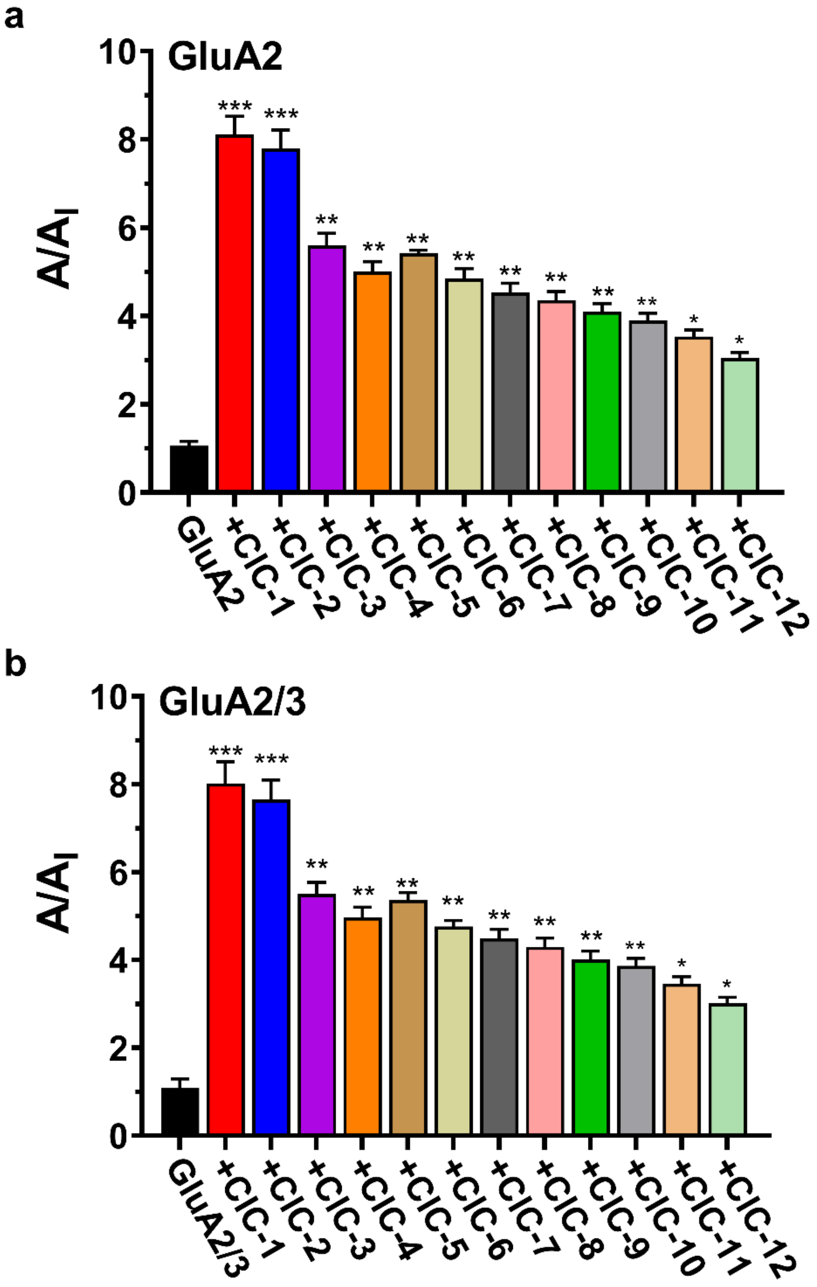

Isoxazole-Carboxamide derivatives spectrum data

All of the following spectrum data were published before [1]

3-(2,6-dichlorophenyl)-5-methyl-N-(4-(methylthio)phenyl)isoxazole-4-carboxamide (CIC-1).

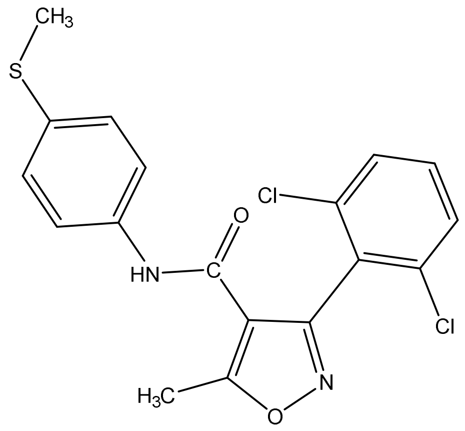

HRMS (ESI): m/z calcd. for $C_{18}H_{14}Cl_2N_2O_2S$ $[M+H]^+$: 393.0231; found: 393.0237. ^{1}H NMR (400 MHz, DMSO$_6$) δ: 10.19 (s, 1H), 7.66-7.60 (m, 2H), 7.59-7.49 (m, 3H), 7.28-7.20 (m, 2H), 2.74 (s, 3H), 2.45 (s, 3H). ^{13}C NMR (101 MHz, DMSO$_6$) δ: 170.15, 159.00, 158.68, 136.49, 134.90, 133.24, 132.71, 128.84, 127.53, 127.42, 121.03, 114.91, 15.92, 13.19.

N-(4-chloro-2,5-dimethoxyphenyl)-3-(2,6-dichlorophenyl)-5-methylisoxazole-4-carboxamide (CIC-2).

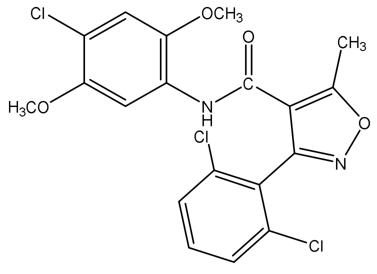

HRMS (ESI): m/z calcd. for $C_{19}H_{15}Cl_3N_2O_4$ $[M+H]^+$: 441.0192; found: 441.0192. ^{1}H NMR (400 MHz, DMSO$_6$) δ:8.32 (s, 1H), 7.99 (s, 1H), 7.78 (d, J = 8.5 Hz, 2H), 7.71 (dd, J = 9.4, 6.8 Hz, 1H), 7.13 (s, 1H), 3.77 (s, 3H), 3.65 (s, 3H), 2.84 (s, 3H). ^{13}C NMR (101 MHz, DMSO$_6$) δ: 174.66, 158.62, 156.89, 148.65, 143.29, 135.26, 133.76, 129.42, 126.52, 126.50, 116.14, 113.55, 112.74, 106.13, 57.15, 56.89, 13.49.

3-(2,6-dichlorophenyl)-N-(3,5-dimethoxyphenyl)-5-methylisoxazole-4-carboxamide (CIC-3).

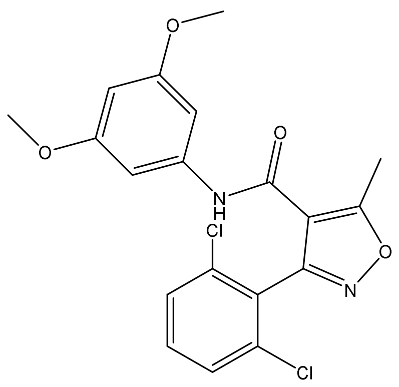

HRMS (ESI): m/z calcd. for $C_{19}H_{16}Cl_2N_2O_4$ $[M+H]^+$: 407.0565; found: 407.0558. ^{1}H NMR (400 MHz, $DMSO_6$) δ: 10.14 (s, 1H), 7.63 (d, *J* = 8.6 Hz, 1H), 7.56 (dd, *J* = 9.3, 6.7 Hz, 1H), 6.83 (s, 2H), 6.26 (s, 1H), 3.71 (s, 6H), 2.73 (s, 3H). ^{13}C NMR (101 MHz, $DMSO_6$) δ: 170.22, 160.94, 159.17, 158.62, 140.78, 134.92, 132.73, 128.86, 127.51, 114.99, 98.46, 96.50, 55.61, 13.18.

3-(2,6-dichlorophenyl)-5-methyl-N-(3,4,5-trimethoxyphenyl)isoxazole-4-carboxamide (CIC-4).

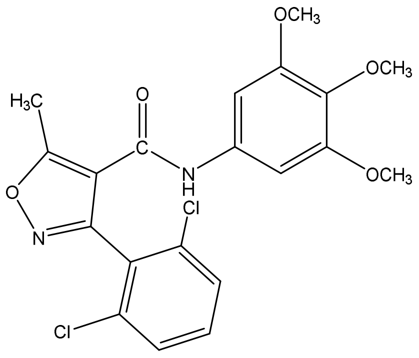

HRMS (ESI): m/z calcd. for $C_{20}H_{18}Cl_2N_2O_5$ $[M+H]^+$: 437.0671; found: 437.0671. ^{1}H NMR (400 MHz, $DMSO_6$) δ: 10.14 (s, 1H), 7.64 (d, *J* = 7.3 Hz, 2H), 7.57 (dd, *J* = 9.2, 6.8 Hz, 1H), 6.98 (s, 2H), 3.73 (s, 6H), 3.62 (s, 3H), 2.73 (s, 3H). ^{13}C NMR (101 MHz, $DMSO_6$) δ: 170.15, 158.96, 158.63, 153.18, 135.32, 134.91, 134.26, 132.74, 128.87, 127.55, 115.02, 97.72, 60.56, 56.16, 13.20.

3-(2,6-dichlorophenyl)-N-(2,4-dimethoxyphenyl)-5-methylisoxazole-4-carboxamide (CIC-5).

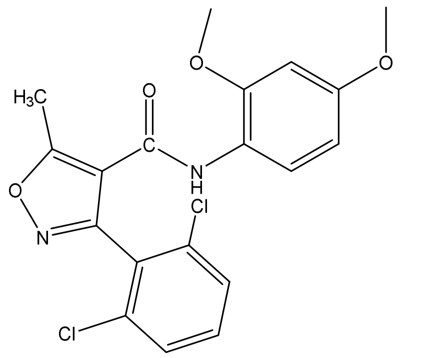

HRMS (ESI): m/z calcd. for $C_{19}H_{16}Cl_2N_2O_4$ $[M+H]^+$: 407.0565; found: 407.0565. ^{1}H NMR (400 MHz, DMSO6) δ: 8.26 (s, 1H), 7.82 (d, *J* = 8.8 Hz, 1H), 7.78-7.58 (m, 3H), 6.59 (d, *J* = 2.6 Hz, 1H), 6.48 (dd, *J* = 8.8, 2.7 Hz, 1H), 3.74 (s, 3H), 3.67 (s, 3H), 2.81 (s, 3H). ^{13}C NMR (101 MHz, DMSO6) δ: 173.46, 158.27, 157.51, 157.17, 151.15, 135.23, 133.48, 129.27, 126.86, 122.86, 120.05, 113.24, 104.66, 99.25, 56.33, 55.82, 13.29.

3-(2,6-dichlorophenyl)-N-(3,4-dimethoxyphenyl)-5-methylisoxazole-4-carboxamide (CIC-6)

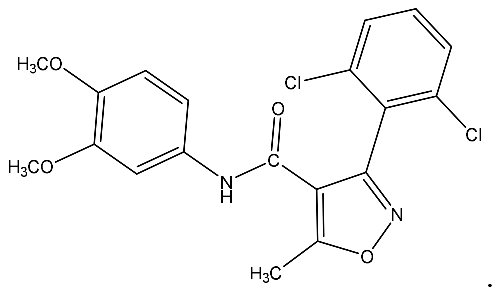

.

HRMS (ESI): m/z calcd. for $C_{19}H_{16}Cl_2N_2O_4$ $[M+H]^+$: 407.0565; found: 407.0564. ^{1}H NMR (400 MHz, DMSO6) δ: 10.04 (s, 1H), 7.63 (dd, *J* = 7.7, 1.5 Hz, 1H), 7.59 – 7.51 (m, 1H), 7.28 (d, *J* = 2.4 Hz, 1H), 7.07 (dd, *J* = 8.6, 2.4 Hz, 1H), 6.89 (d, *J* = 8.7 Hz, 1H), 3.72 (s, 3H), 3.71 (s, 3H), 2.74 (s, 3H). ^{13}C NMR (101 MHz, DMSO6) δ: 169.96, 158.76, 158.65, 149.00, 145.84, 134.93, 132.67, 132.65, 128.83, 127.65, 115.05, 112.47, 112.29, 105.42, 56.24, 55.87, 13.16.

3-(2,6-dichlorophenyl)-N-(4-(2-methoxyphenoxy)phenyl)-5-methylisoxazole-4-carboxamide (CIC-7).

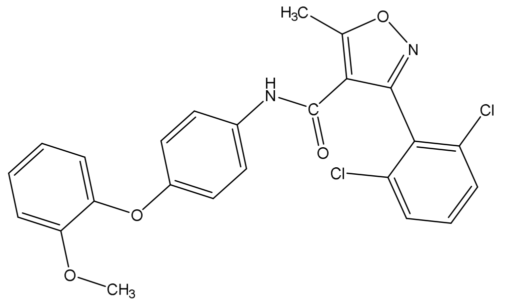

HRMS (ESI): m/z calcd. for $C_{24}H18Cl_2N_2O_4$ $[M+H]^+$: 469.0722; found: 469.0711. ^1H NMR (400 MHz, DMSO6) δ: 10.12 (s, 1H), 7.61 (dd, *J* = 6.6, 3.2 Hz, 1H), 7.55 (d, *J* = 3.2 Hz, 1H), 7.48 (d, *J* = 9.0 Hz, 1H), 7.16 (d, *J* = 3.4 Hz, 1H), 7.03–6.86 (m, 1H), 6.85–6.77 (m, 1H), 3.74 (s, 3H), 2.74 (s, 3H). ^{13}C NMR (101 MHz, DMSO6) δ: 170.02, 158.90, 158.69, 154.44, 151.67, 144.44, 134.92, 133.58, 132.65, 128.81, 127.62, 125.78, 122.13, 121.61, 121.54, 116.92, 114.94, 113.85, 56.08, 13.15.

3-(2,6-dichlorophenyl)-5-methyl-N-phenylisoxazole-4-carboxamide (CIC-8).

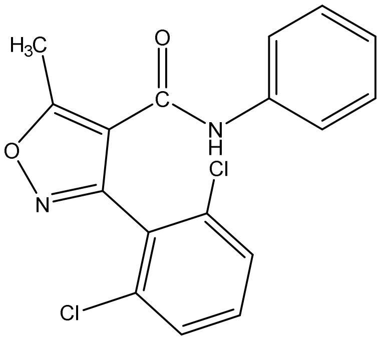

HRMS (ESI): m/z calcd. for $C_{17}H_{12}Cl_2N_2O_2$ $[M+H]^+$:347.0354; found: 347.0354. ^1H NMR (400 MHz, $DMSO_6$) δ: 10.18 (s, 1H), 7.64 (d, *J* = 1.8 Hz, 1H), 7.61 (d, *J* = 2.8 Hz, 1H), 7.59 – 7.55 (m, 1H), 7.32 (t, *J* = 7.1 Hz, 2H), 7.16 – 7.07 (m, 1H), 2.75 (s, 3H). ^{13}C NMR (101 MHz, $DMSO_6$) δ: 170.16, 159.13, 158.69, 139.05, 134.93, 132.69, 129.18, 128.83, 127.58, 124.43, 120.42, 114.96, 13.18.

N-([1,1′-biphenyl]-4-yl)-3-(2,6-dichlorophenyl)-5-methylisoxazole-4-carboxamide (CIC-9)

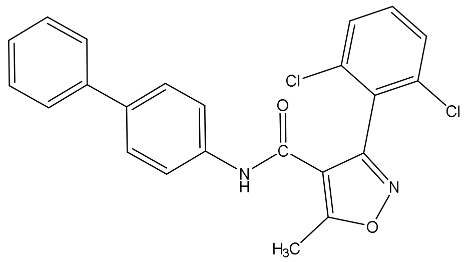

HRMS (ESI): m/z calcd. for $C_{23}H_{16}Cl_2N_2O_2$ $[M+H]^+$: 423.0667; found: 423.0673. ^1H NMR (400 MHz, $DMSO_6$) δ: 10.30 (s, 1H), 7.74-7.60 (m, 8H), 7.60-7.52 (m, 1H), 7.50-7.41 (m, 2H), 7.38-

7.31 (m, 1H), 2.77 (s, 3H). ^{13}C NMR (101 MHz, DMSO$_6$) δ: 170.21, 159.15, 158.70, 140.10, 138.54, 136.15, 134.93, 132.71, 129.38, 128.85, 127.62, 127.55, 127.40, 126.79, 120.72, 114.98, 13.20.

3-(2,6-dichlorophenyl)-5-methyl-N-(4-(trifluoromethoxy)phenyl)isoxazole-4-carboxamide (CIC-10)

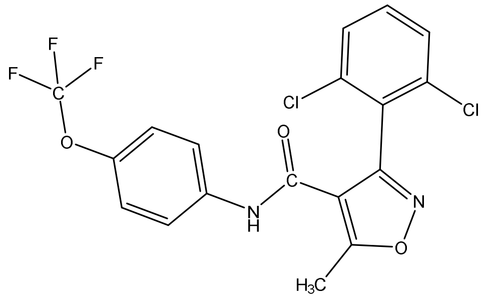

HRMS (ESI): m/z calcd. for $C_{18}H_{11}Cl_2F_3N_2O_3$ [M+H]$^+$: 431.0177; found: 431.0175. ^1H NMR (400 MHz, DMSO6) δ: 10.41 (s, 1H), 7.71 – 7.66 (m, 1H), 7.65 – 7.61 (m, 1H), 7.59 – 7.53 (m, 1H), 7.33 (d, *J* = 8.6 Hz, 2H), 2.76 (s, 3H). ^{13}C NMR (101 MHz, DMSO6) δ: 170.41, 159.31, 158.68, 144.51, 138.25, 134.90, 132.73, 128.84, 127.45, 122.10, 121.83, 114.73, 13.18.

N-(4-(tert-butyl)phenyl)-3-(2,6-dichlorophenyl)-5-methylisoxazole-4-carboxamide (CIC-12).

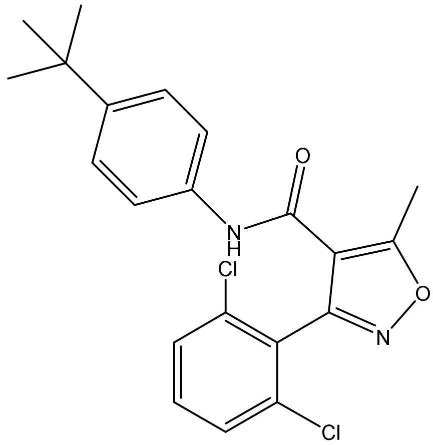

HRMS (ESI): m/z calcd. for $C_{21}H_{20}Cl_2N_2O_2$ [M+H]$^+$: 403.0833; found: 403.0835. ^1H NMR (400 MHz, DMSO$_6$) δ: 10.11 (s, 1H), 7.68-7.52 (m, 3H), 7.55-7.43 (m, 3H), 7.32 (d, *J* = 8.6 Hz, 2H), 2.73 (s, 3H), 1.26 (s, 9H). ^{13}C NMR (101 MHz, DMSO$_6$) δ: 170.03, 158.98, 158.67, 146.80, 136.53, 134.90, 132.68, 128.83, 127.58, 125.80, 120.09, 115.04, 34.52, 31.62, 13.14.

1. Hawash, M., et al., *Exploration of isoxazole analogs: Synthesis, COX inhibition, anticancer screening, 3D multicellular tumor spheroids, and molecular modeling.* European Journal of Medicinal Chemistry, 2024. **271**: p. 116397.
